# Direct Imaging of Carrier Funneling in a Dielectric Engineered 2D Semiconductor

**DOI:** 10.1021/acsnano.3c05957

**Published:** 2023-12-18

**Authors:** Nicolas Gauriot, Arjun Ashoka, Juhwan Lim, Soo Teck See, Jooyoung Sung, Akshay Rao

**Affiliations:** † Cavendish Laboratory, University of Cambridge, CB3 0HE Cambridge, United Kingdom; ‡ Department of Physics and Chemistry, DGIST, Daegu 42988, Republic of Korea

**Keywords:** two-dimensional materials, microscopy, transient absorption, transition-metal dichalcogenides, transport, excitons

## Abstract

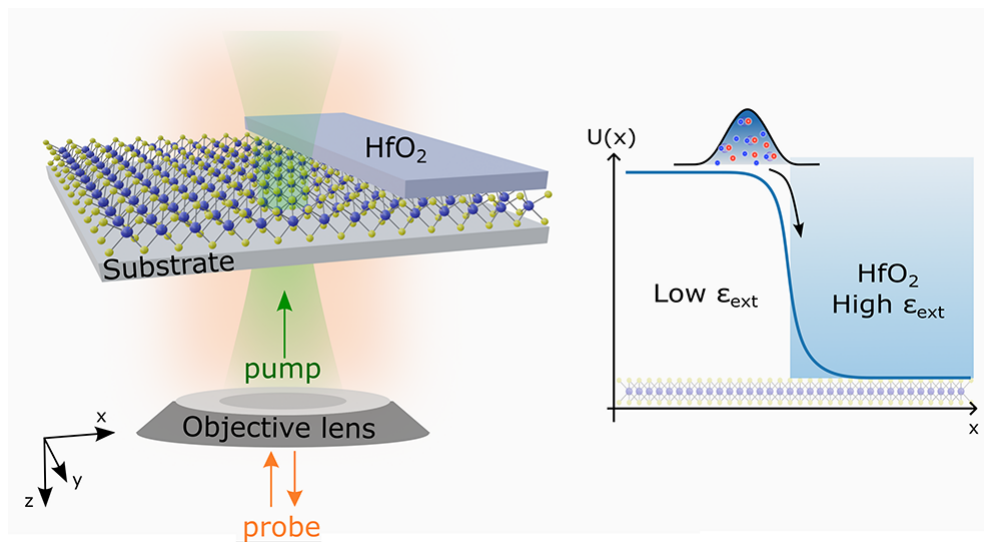

In atomically thin transition-metal dichalcogenides (TMDCs), the environmental sensitivity of the strong Coulomb interaction offers promising approaches to create spatially varying potential landscapes in the same continuous material by tuning its dielectric environment. Thus, allowing for control of transport. However, a scalable and CMOS-compatible method for achieving this is required to harness these effects in practical applications. In addition, because of their ultrashort lifetime, observing the spatiotemporal dynamics of carriers in monolayer TMDCs, on the relevant time scale, is challenging. Here, we pattern and deposit a thin film of hafnium oxide (HfO_2_) via atomic layer deposition (ALD) on top of a monolayer of WSe_2_. This allows for the engineering of the dielectric environment of the monolayer and design of heterostructures with nanoscale spatial resolution via a highly scalable postsynthesis methodology. We then directly image the transport of photoexcitations in the monolayer with 50 fs time resolution and few-nanometer spatial precision, using a pump probe microscopy technique. We observe the unidirectional funneling of charge carriers, from the unpatterned to the patterned areas, over more than 50 nm in the first 20 ps with velocities of over 2 × 10^3^ m/s at room temperature. These results demonstrate the possibilities offered by dielectric engineering via ALD patterning, allowing for arbitrary spatial patterns that define the potential landscape and allow for control of the transport of excitations in atomically thin materials. This work also shows the power of the transient absorption methodology to image the motion of photoexcited states in complex potential landscapes on ultrafast time scales.

Controlling the transport of electronic excitations is central to semiconductor technologies. To this end, several strategies have been developed to tailor the band structure of materials on the nanoscale. This includes spatial variations in the material stoichiometry,^1^ strain gradients,^2^ or the formation of a heterojunction with different materials.^3^

Compelling opportunities in band structure design have appeared with the advent of two-dimensional (2D) semiconductors. Because they are atomically thin, they are very sensitive to their environment. For example, strain,^4−14^ environmental dielectric screening,^15−25^ polarization of ferroelectric substrate,^26,27^ and adsorption of chemicals on the surface^28,29^ have been shown to have a significant impact on the energetics of the material. This offers the possibility to generate complex potential landscapes, postsynthesis and in the same material, by engineering the material’s surroundings.^8,11,14,15,20,26,27,30,31^

Among 2D semiconductors, monolayer (ML) transition-metal dichalcogenides (TMDCs), are particularly promising for optoelectronic applications thanks to the combination of direct bandgaps, large carrier mobilities, and strong light–matter interaction.^32−35^ The optical response of a ML TMDC is dominated by excitons because of an unusually strong Coulomb interaction.^36,37^ The origin of this strong Coulombic interaction is well understood. The electrostatic potential of a charge is screened more or less effectively, depending on the permittivity of the medium in which it is embedded in. For an atomically thin material, this includes the surrounding media, which is usually a low-dielectric material (typically air). The screening of the Coulomb interaction is therefore much lower than in bulk inorganic semiconductors. This also means that the electron–hole interaction and the electron–electron interaction depend on the dielectric constant of the surrounding media.^15−19,21,22,38^ Consequently, both the quasiparticle bandgap and the exciton binding energy can be tuned by engineering the local dielectric environment.

Although the consequences of strain gradients on excitonic transport have been extensively studied,^6,8,11,13,14,39−43^ the full possibilities offered by dielectric engineering remain underexplored. Specifically, two experimental challenges must be overcome. First, we need scalable ways to pattern the dielectric environment over large areas, rather than simply exfoliating and stacking small areas of arbitrarily shaped graphene or h-BN onto the 2D material to be engineered, as has been done to date. Second, we require tools to understand the dynamics and transport of photoexcitation in these complex potential landscapes. This has been challenging to date, as most studies rely on photoluminescence (PL) microscopy, which lacks both the ultrafast time resolution and the nanoscale spatial precision to study these phenomena on the relevant spatiotemporal scales. Furthermore, PL is insensitive to dark excitons and charges, both of which play major roles in these materials.

Here, we tackle both of these issues and demonstrate the patterning of a WSe_2_ monolayer with atomic layer deposition, which in principle allows for arbitrary spatial patterns via a highly scalable methodology. We use this method to pattern areas covered with HfO_2_, a high-dielectric-constant insulating material which significantly alters the dielectric environment around the WSe_2_ monolayer. Then, using fs-transient optical absorption microscopy, we directly image the motion of carriers, with high temporal resolution and spatial precision well below the diffraction limit. We observe the unidirectional funneling of charge carriers, from the unpatterned to the patterned areas, over more than 50 nm in the first 20 ps after photoexcitations, with velocities over 2 × 10^3^ m/s at room temperature.

## Results and Discussion

**Figure 1:**
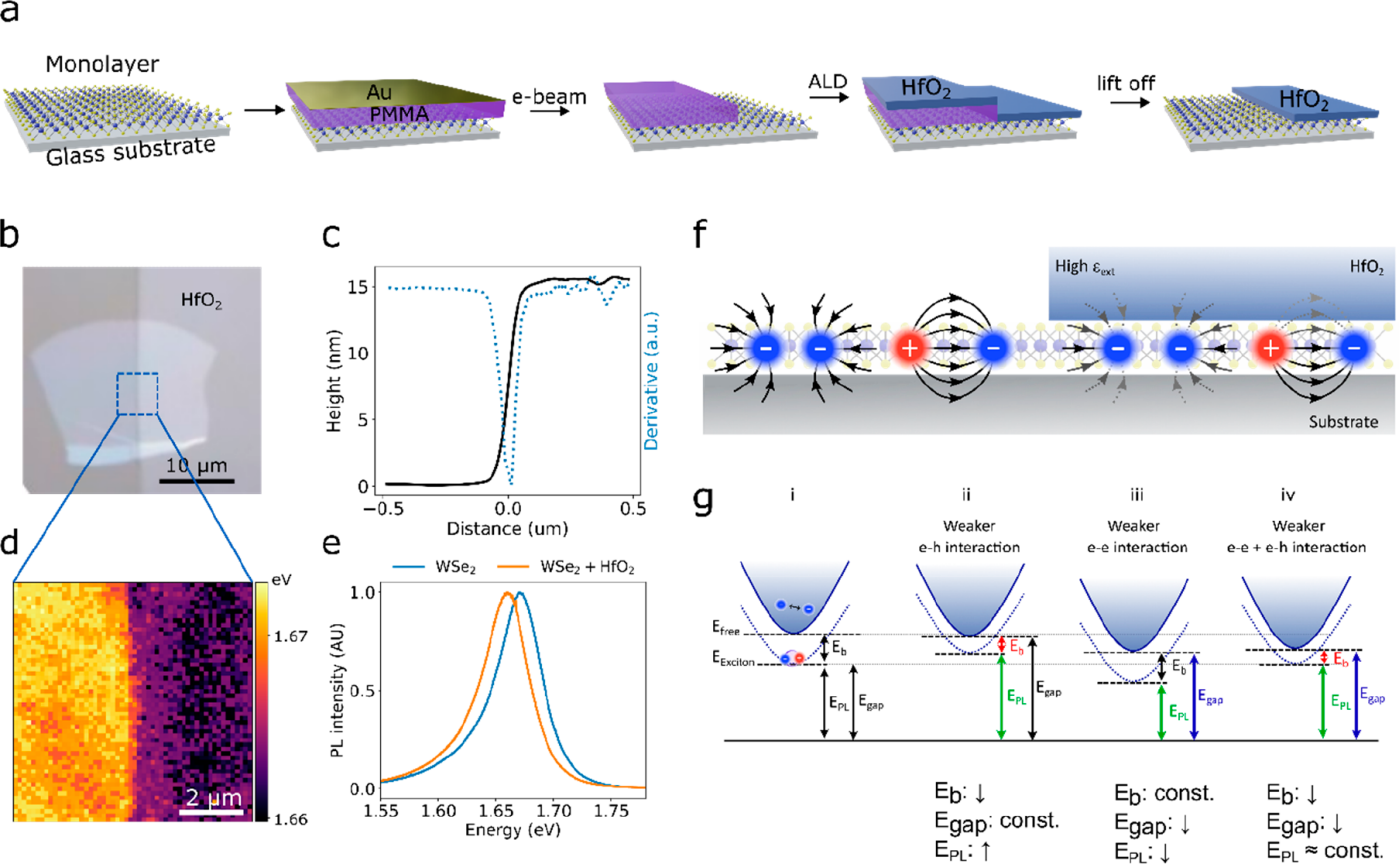
Coulomb engineering in monolayer WSe_2_. (a) Schematic of the heterostructure fabrication process. (b) Micrograph of a representative sample. (c) AFM height profile (black) and its derivative (blue) across the junction. (d) Map of the transition energy extracted from PL spectra. (e) Representative photoluminescence spectra on each side of the junction. (f) Schematic of the sample architecture. (g) Schematic of the energy levels and the effect of the different interaction terms on the different electronic transitions (see main text for details).

Figure 1b shows a micrograph of a representative sample: a monolayer of WSe_2_ partially covered by a 15 nm thick HfO_2_ film. We chose HfO_2_ for its high dielectric constant (ε ≈ 25),^44^ and its wide use as a gate oxide in CMOS-compatible technologies. Here, the HfO_2_ patch is patterned by e-beam lithography and deposited by ALD as described in detail in Figure 1a. Figure 1c presents an AFM height profile of the junction and its derivative, with a fwhm of ∼50 nm, illustrating the sharpness of the junction (a full AFM map of the junction is presented in Supplementary Note 1). Notably, unlike methods relying on the stamping of materials on the 2D layer, this process is highly scalable and could be easily extended to generate more complex patterns.

In order to understand the effect of the high-dielectric environment on the electronic structure of WSe_2_, we performed scanning confocal photoluminescence (PL) measurements on both the pristine WSe_2_ monolayer and the WSe_2_/HfO_2_ stack. Representative PL spectra from each side of the junction are presented in Figure 1e. We then extract the energy of the optical gap from the PL spectra obtained over the junction and retrieve the 2D map of the transition energy, as shown in Figure 1d. We observe a clear 17 meV red shift of the excitonic transition on the HfO_2_-covered side compared to the pristine layer.

Since HfO_2_ has a large dielectric constant, the screening of the electron–hole interaction in WSe_2_ is more effective on the side covered by HfO_2_, which reduces the binding energy and blue-shifts the exciton transition (Figure 1g(ii). At the same time, since the electron–electron interaction is also weaker, the bandgap is reduced. This red-shifts the transition (Figure 1g(iii). Based on previous experimental and theoretical work, we expect the decrease in quasiparticle gap and in the exciton binding energy, on the HfO_2_-covered side, to be of similar magnitude and on the order of 100 meV.^15,21,25,45^ These two opposite effects almost cancel each other, and in the end, the optical gap is only modified by a small amount (Figure 1g(iv)). We note that the sharp shift in the optical gap is observed only after annealing the sample (see Methods). Prior to annealing, we also observe a red shift of the PL on the HfO_2_-covered flake; however the shifts are larger and not homogeneous and the transition from pristine to covered flake is not sharp (see Supplementary Note 2). This suggests that the ALD process induces strain in the monolayer leading to additional shifts of the optical gap.^46^ Thermal annealing relaxes strain, and a sharp in-plane junction that strictly follows the HfO_2_ patch is created.

**Figure 2:**
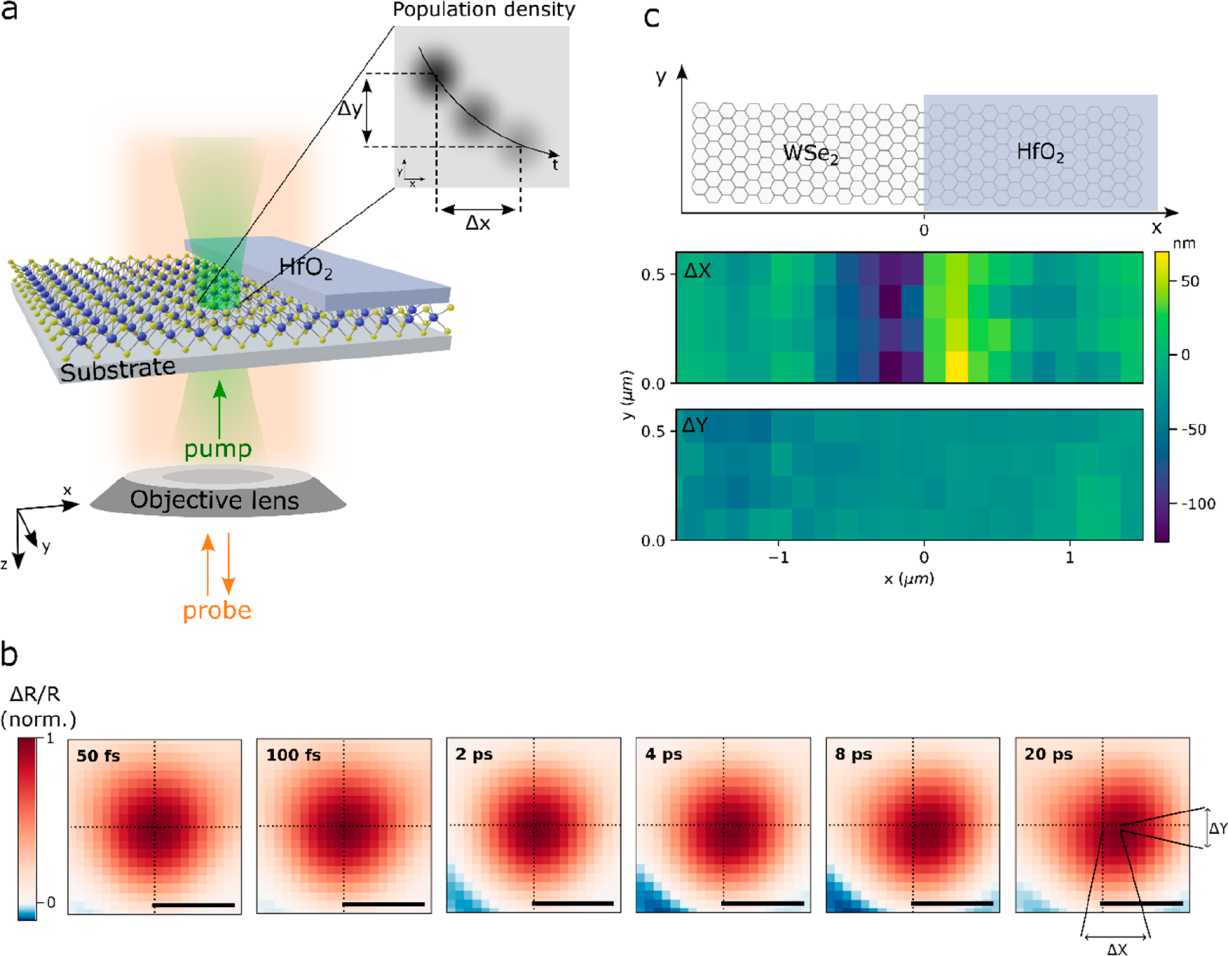
Imaging of the ultrafast transport. (a) Schematic of the pump–probe microscopy experiment. See main text for details. (b) Time evolution of the transient reflection signal after excitation at a representative point near the boundary. (c) Map of the extracted shifts of the excited state population in the direction perpendicular to the junction (Δ*X*) and parallel to the junction (Δ*Y*) after a 5 ps delay, as a function of the excitation position. The boundary is located at *x* = 0 as illustrated in the top panel.

To image the transport of excitations in this asymmetric potential landscape, we use a transient reflection microscopy technique. Figure 2a presents a schematic of the experiment. Briefly, a 15 fs pump pulse, focused near the diffraction limit, generates a population of excited states. After a controllable time delay, the transient reflection of the sample is imaged onto a camera with a wide-field probe pulse. More details on the experimental setup are presented in Methods and elsewhere.^47,100^ In contrast to PL-based imaging, our technique is sensitive to both bright and dark states, and because it relies on optical gating, it exhibits a much faster time resolution. As previously described, differential imaging allows tracking the population of photoexcitations in the material, in time and space, with 50 fs time resolution and few-nanometer precision.^47,100^Figure 2b shows the evolution of the transient reflection (Δ*R*/*R*) images obtained after exciting the sample at a representative point near the junction where we can clearly see the movement over time of the center of mass of the Δ*R*/*R* signal.

In order to quantify this motion, we extract the position of the excited state distribution (*X*(*t*), *Y*(*t*)) at each time delay by fitting these images with a 2D Gaussian distribution, and we measure the movement of the distribution in the direction perpendicular (Δ*X*(*t*) = *X*(0) – *X*(*t*)) and parallel (Δ*Y*(*t*) = *Y*(0) – *Y*(*t*)) to the junction as illustrated in the inset of Figure 2a. To study how this motion varies depending on where the excitations are launched (i.e., the position at *t* = 0), we repeat this experiment at various points around the junction. For each starting position (*X*(0),*Y*(0)) we extract the Δ*X* and Δ*Y* shifts after a 5 ps delay to build up the maps presented in Figure 2c. We observe no significant shift (Δ*Y*) of the mean of the distribution in the direction parallel to the junction irrespective of the excitation position, while in the perpendicular direction (Δ*X*), we measure clear positive and negative shifts for the starting position in the vicinity of the junction. This observation of opposite Δ*X* shifts on each side of the junction paints a slightly more complex situation than a simple unidirectional funneling of excitations across the junction, and a more detailed analysis is required before conclusions on transport can be drawn.

**Figure 3:**
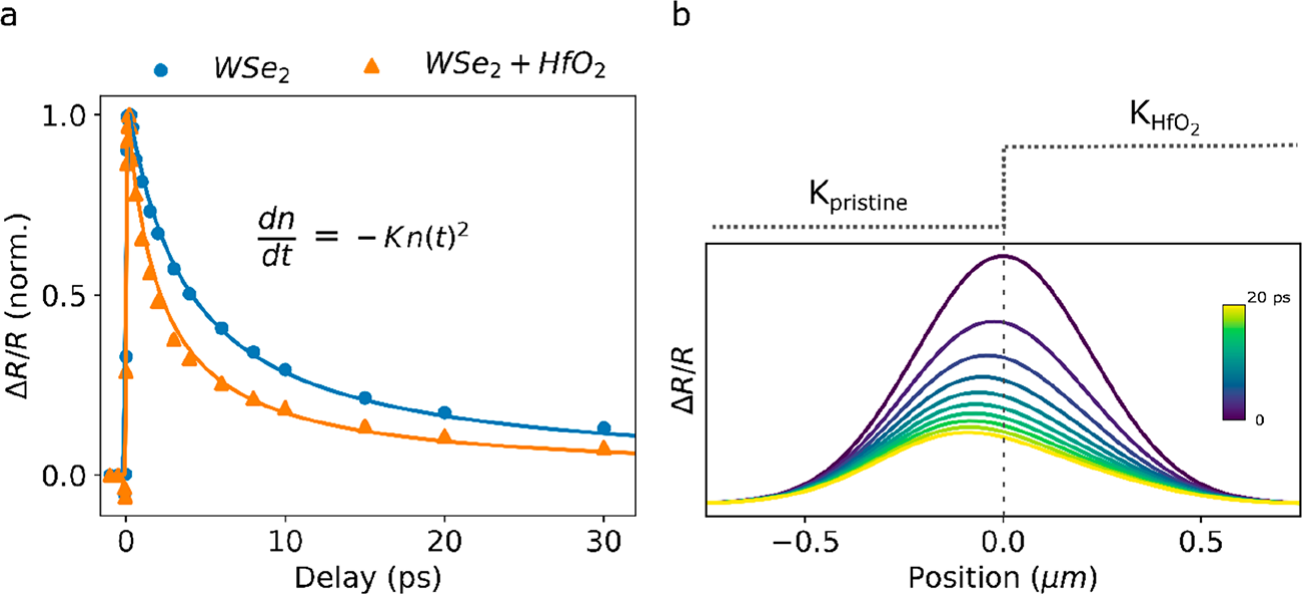
Effect of the spatially varying kinetics. (a) Decay kinetics of the Δ*R*/*R* signal far from the boundary on the pristine side (blue dots) and the HfO_2_-covered side (orange triangles). Solid lines are fits to a bimolecular decay, describing exciton–exciton annihilation. (b) Simulated Gaussian profile decaying through a bimolecular decay with a spatially dependent decay rate. The decay rate follows the step function illustrated in the top panel with values of *K*_WSe_2__ and *K*_HfO_2__ extracted from (a). The profiles are convoluted with a Gaussian to simulate the point spread function of the microscope.

To understand the origin of these opposite Δ*X* shifts, we measure the decay dynamics on each side of the junction far from the boundary. As shown in Figure 3a, the two kinetics are well approximated by a bimolecular decay, with a decay rate *k*_HfO_2__ = 1.7 × 10^–2^ cm^2^ s^–1^ on the covered side and *k*_pristine_ = 0.9 × 10^–2^ cm^2^ s^–1^ on the pristine side. This is consistent with previous studies showing that the decay dynamics in TMDC monolayers are dominated by an efficient exciton–exciton annihilation.^48−50^ Since the Δ*R*/*R* signal decays faster on the HfO_2_ side than on the pristine side, in the absence of any transport, a Gaussian distribution sitting across the junction will appear to move toward the pristine side (Δ*X* < 0), as illustrated in Figure 3b. This means that the negative shifts do not reflect a movement of the population but rather a change in its spatial profile due to the underlying difference in the decay dynamics on either side of the junction. The measured spatial shifts are therefore due to a combination of two effects: actual transport of excitons in the monolayer and a spatial variation of the decay dynamics. Consequently, the raw Δ*X* shifts presented here are smaller than the true motion of the excitations in the monolayer. More generally, the raw spatial shifts measured in time-resolved microscopy experiments similar to that presented here (whether based on pump–probe or photoluminescence signals) should not be interpreted as movement of carriers in the material without a spatially resolved analysis of the decay dynamics.

**Figure 4:**
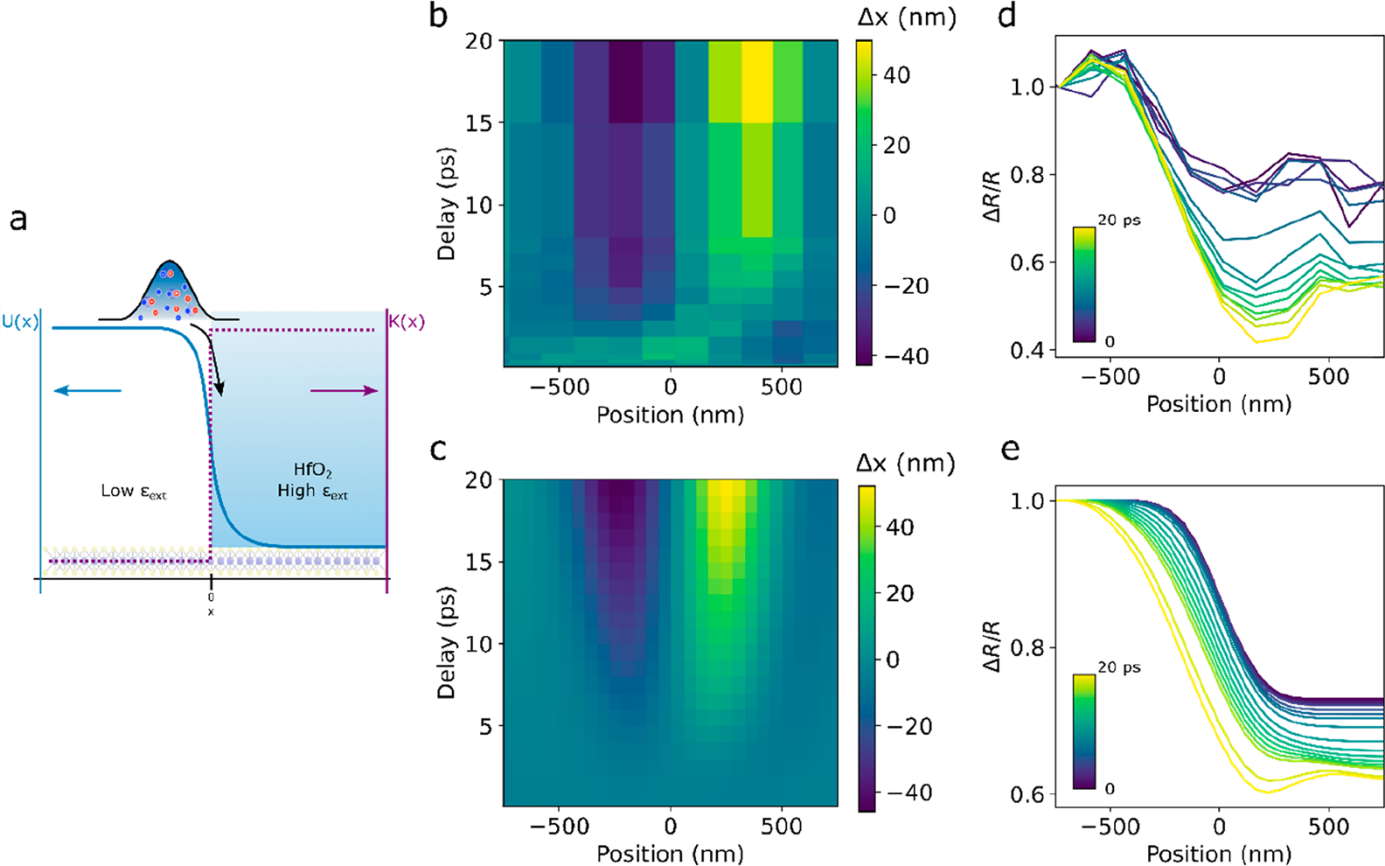
Unidirectional exciton funnelling. (a) Schematic of the modeled potential step *U* and annihilation rate *K* across the junction. (b) Map of the Δ*X* shift as a function of time and excitation position near the junction. (c) Corresponding simulated map. (d) Temporal variation of Δ*R*/*R* corresponding to the map in (b). (e) Corresponding simulated Δ*R*/*R*.

In order to evaluate the actual transport of excitations in the monolayer, we modeled the spatiotemporal evolution of the excited state population across the junction with a drift diffusion model. Considering the excitons as quasiparticles evolving in a potential landscape *U*(*x*) and decaying through a bimolecular recombination with spatially varying decay rate *k*(*x*), the exciton population *n*(*r*,*t*) is described by the equation
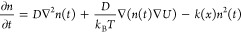
where *D* is the diffusion coefficient in the monolayer. We model *U* as a smooth step function, , where erf is the error function, such that ∇*U* is a Gaussian of variance σ, while *k*(*x*) = *k*_pristine_ + *H*(*x*){*k*_pristine_ – k_HfO_2__}, with *H* being the Heaviside step function, as illustrated in Figure 4a. Importantly, we fix the annihilation rate *k*_pristine_ and k_HfO_2__ to the values extracted from Figure 3. Next, we model the initial exciton distribution as a Gaussian such that , and to simulate the microscope objective point spread function (PSF) the calculated *n*(*r*,*t*) distribution is convolved with another Gaussian, . Finally, we extract the position *X*(*t*) of the simulated distribution with the same analysis as that with the measured data described before. Figure 4 compares the measured and simulated data. Figure 4b shows a map of the measured Δ*X* shift as a function of time and initial position around the junction. Figure 4d presents the time evolution of the amplitude of the measured Δ*R*/*R* signal at the same positions. Figure 4c,e shows the corresponding simulation results, in good agreement with the experimental results, for *D* = 2 cm^2^/s, *U*_0_ = 150 meV, and σ = 55 nm. This simple model therefore confirms the funneling and unidirectional motion of excitations from the pristine side to the HfO_2_-covered side of the junction. We note that varying *D* from 0.1 to 10 cm^2^/s but keeping the drift current *J* = D/k_B_T ∇(n(t)∇U) constant (i.e., keeping *DU*_0_ constant) does not affect the simulation results significantly (see Supplementary Note 3). This suggests that for the time scale studied here (<20 ps) the effect of the diffusion term is negligible compared to the drift current.

The optical pump used here is centered at 550 nm (2.25 eV) and is well above the free particle gap of monolayer WSe_2_. The pump fluence is 17 μJ/cm^2^, which with an absorption of 10%^51^ leads to 4 × 10^12^/cm^2^ absorbed photons. Under these conditions, mid-infrared pump probe spectroscopy measurements revealed that excitons form within few hundreds of fs, but about 40% of excitations remain as unbound electrons and holes.^52^

Reported values of diffusion coefficients for excitons in monolayer TMDC range from *D* = 0.1 cm^2^/s to *D* = 10 cm^2^/s.^48,53−57^ However, even if we assume the largest diffusion constant, a potential step *U*_0_ = 30 meV is required to match the experimental data, and the 17 meV potential step of the excitons, which is lower than the thermal energy (*k*_B_*T* ≈ 25 meV), is too small to drive the observed motion across the junction. Free charges, however, with a potential step across the junction given by the change in quasiparticle gap of about 100 meV are more likely to be driving this motion. Reported mobilities in TMDC are on the order of 100 V cm^2^/s,^33,58^ which leads to the diffusion coefficient *D* ≈ 2.5 cm^2^/s, bringing *U*_0_ to 120 meV, in good agreement with the expected change in the free particle gap across the junction.

## Conclusion

To summarize, by making use of the sensitivity to the dielectric environment of monolayer TMDC, we have engineered monolayer WSe_2_. We have observed a unidirectional flow of excitations through the junction from the unpatterned to the patterned areas, with shifts of the mean position of excitation higher than 50 nm in 20 ps, leading to a lower bound for the carrier velocity of 2.5 × 10^3^ m/s. This simple proof of concept showcases the opportunity offered by 2D semiconductors to engineer potential landscapes to direct excitations and reveals the power of transient absorption microscopy as a technique to observe energy flows in materials on an ultrafast time scale.

## Methods

### Sample Preparation

Monolayers of WSe_2_ were exfoliated from bulk crystal (HQgraphene) on PDMS films (TELTEC) and transferred on a 170 nm thick glass substrate with a dry transfer technique.^59^ Electron beam lithography was used to pattern the HfO_2_ film. 15 nm of gold was evaporated on top of the PMMA resist to act as a discharge layer during the lithography and etch after exposure (gold etchant standard, Sigma-Aldrich). A 15 nm film of HfO_2_ was then grown with atomic layer deposition (125 °C, HfCl_4_ and H_2_O precursors) before lift-off in acetone. The samples were subsequently annealed in at 200 °C in partial vacuum for 2 h to relax strain.

### Transient Absorption Microscopy

Our pump–probe microscopy setup was described previously.^47,100^ Here, a pump pulse (centered at 550 nm, fluence 17 μJ/cm^2^) is focused onto the sample with an oil immersion objective (NA = 1.1) to produce a near-diffraction-limited local photoexcitation. After a variable time delay, a copropagating wide-field probe pulse (750 nm, ∼20 μm full width at half-maximum) is reflected on the sample and imaged onto an emCCD camera (Rolera Thunder, QImaging). Wide-field probe images in the presence and absence of the pump excitation are recorded by chopping the pump pulse at 40 Hz. The pump and probe pulses are derived from a Yb:KGW amplifier (1030 nm, 5 W, 200 kHz, 200 fs, LightConversion) via white-light continuous generation and subsequent spectral filtering and compression with chirped mirrors. After transmission through the objective and the glass substrate, the pump is 13 fs, while the probe is 7 fs before the objective. At the sample the probe is significantly chirped, and integrating the signal over a 10 nm spectral bandwidth gives a 50 fs temporal resolution as demonstrated previously.^47^

### Microphotoluminescence

PL spectroscopy was performed on a Renishaw Invia confocal setup equipped with a motorized piezo stage, using an air-cooled Ar-ion 514.5 nm continuous wave (CW) laser via a 100× objective (NA = 0.9).

## Data Availability

The data underlying all figures in this article are publicly available from the University of Cambridge repository at (https://doi.org/10.17863/CAM.104577).

## Supporting Information Available

AFM height map of the heterostructure, map of the transition energy extracted from PL spectra for a nonannealed sample, and comparison of different simulation results (PDF)
