# Supplementary material for: Direct Imaging of Carrier Funneling in a Dielectric Engineered 2D Semiconductor
Source: ACS Nano. 2023 Dec 18;18(1):264–71. doi: 10.1021/acsnano.3c05957 (PMC10786151; doi:10.1021/acsnano.3c05957)
Supplement: Supplementary file 1 — nn3c05957_si_001.pdf [file nn3c05957_si_001.pdf]

Supporting information

Direct imaging of carriers funnelling in a dielectric engineered 2D semiconductor

Nicolas Gauriot[1], Arjun Ashoka[1], Juhwan Lim[1], Soo Teck See[1], Jooyoung Sung[1,2], Akshay Rao[1*]

[1]Cavendish Laboratory, University of Cambridge, CB3 0HE, Cambridge, United Kingdom

[2]Department of Physics and Chemistry, DGIST, Daegu, 42988, Republic of Korea

Correspondance : ar525@cam.ac.uk

Supplementary Note 1

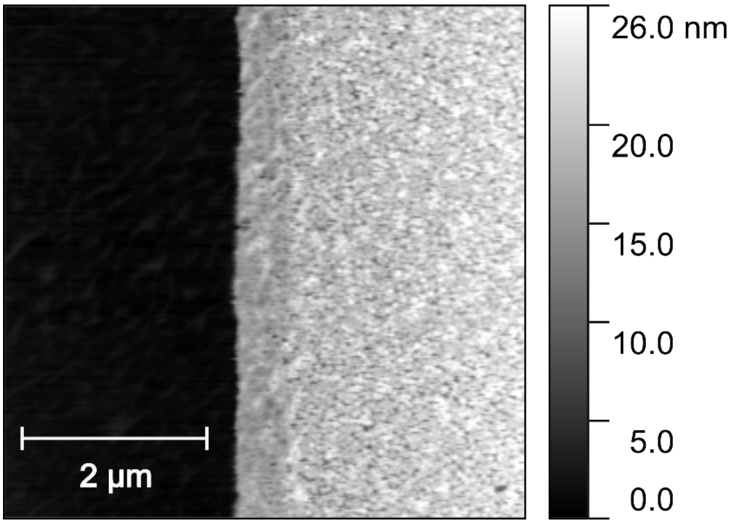

Supplementary Figure 1: AFM height map of the heterostructure, measured in tapping mode.

Supplementary Note 2

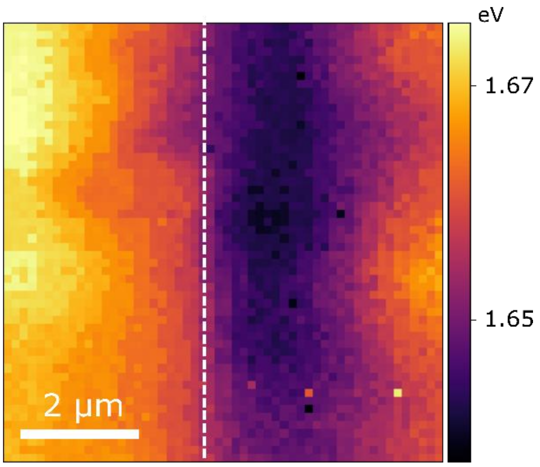

Supplementary Figure 2: Map of the transition energy extracted from PL spectra for a non-annealed sample. Dotted line represents the edge of the HfO2 patch. Compared to Figure 1.d the shift in the transition energy across the junction is larger, smoother, and less homogeneous. This suggest that strained induced by the ALD deposition is relaxed by annealing.

Supplementary Note 3

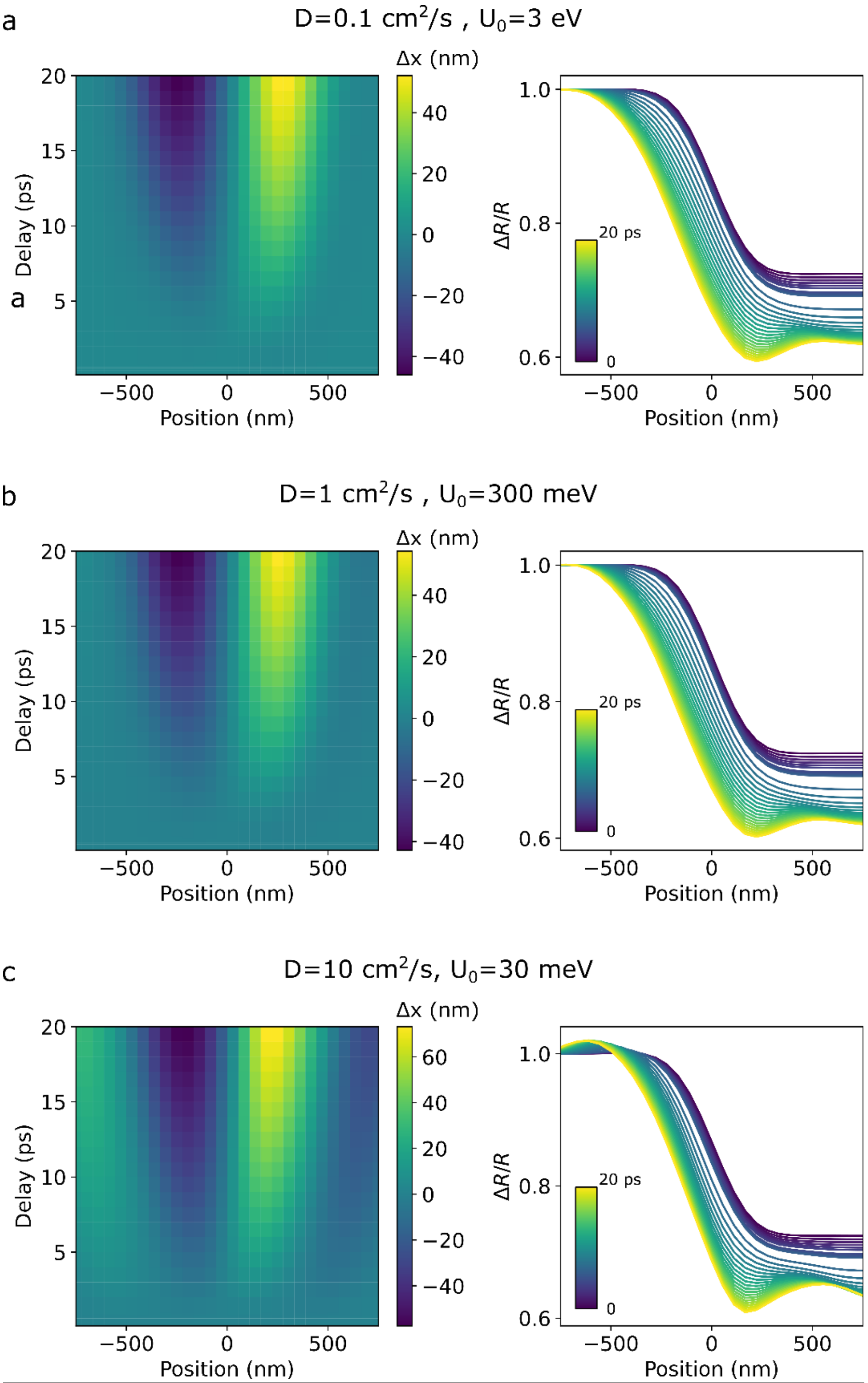

Supplementary Figure 3: Comparison between of the simulation results for different diffusion constants D but keeping D ∇U constant. Left: Map of the Δx shift, right: time evolution of ΔR/R for (a) D = 0.1 cm2/s and U_0 = 3 eV (b) D = 0.1 cm2/s and U_0 = 300 meV and (c) D = 10 cm2/s and U_0 = 30 meV.
